# Deep Learning Prediction of Childhood Myopia Progression Using Fundus Image and Refraction Data

**DOI:** 10.1001/jamanetworkopen.2025.53543

**Published:** 2026-01-26

**Authors:** Meng-Tian Kang, Yansong Hu, Ningli Wang, Jing Fu, Ankang Zhou, Yuanyuan Liu, Hongbei Meng, Xuemeng Li, Shengbo Wang, Xuhang Chen, Hubin Zhao, Guohua Hu, Wei Wang, Yanning Dai, Arokia Nathan, Peter Smielewski, Shuo Gao, Shi-Ming Li

**Affiliations:** 1 Beijing Tongren Eye Center, Beijing Tongren Hospital, Beijing Institute of Ophthalmology, Beijing Key Laboratory of Intelligent Diagnosis Technology and Equipment for Optic Nerve-Related Eye Diseases, National Engineering Research Center for Ophthalmology, Engineering Research Center of Ophthalmic Equipment and Materials (Ministry of Education), Capital Medical University, Beijing, China; 2 School of Instrumentation and Optoelectronic Engineering, Beihang University, Beijing, China; 3 School of Mechanical Engineering, Beihang University, Beijing, China; 4 Division of Neurosurgery, Department of Clinical Neurosciences, University of Cambridge, Cambridge, United Kingdom; 5 Division of Surgery and Interventional Science, University College London, Stanmore, United Kingdom; 6 Department of Electronic Engineering, The Chinese University of Hong Kong, Shatin, N. T., Hong Kong SAR, China; 7 AI Medical Image Division, Thorough Future Inc, Beijing, China; 8 Centers of Excellence, King Abdullah University of Science and Technology, Thuwal, Saudi Arabia; 9 Department of Engineering, University of Cambridge, Cambridge, United Kingdom

## Abstract

**Importance:**

Childhood myopia is a global health concern with escalating prevalence and can lead to severe irreversible visual impairment. Early prediction of myopia progression is crucial for timely intervention to prevent high myopia and associated complications.

**Objective:**

To develop and validate a quantitative method, based on a deep learning method and using only fundus images and baseline refraction data, to predict both myopia progression trajectory and high myopia risk in schoolchildren.

**Design, Setting, and Participants:**

This longitudinal school-based cohort study (Anyang Childhood Eye Study) was conducted from February 2012 to May 2018, with annual follow-up examinations from February 2013 to May 2018. Grade 1 students, aged 6 to 9 years, were recruited from 11 randomly selected urban primary schools in Anyang, Henan Province, China. Children who received myopia control treatments, had amblyopia, or underwent strabismus surgery were excluded. Two independent external validation cohorts were used: the Lhasa cohort (predominantly consisting of Tibetan children) and the Beijing cohort (predominantly consisting of Han Chinese children). Data analysis was performed from July 2024 to July 2025.

**Main Outcomes and Measures:**

Performance of a novel deep learning model, created from combining convolutional neural network (34-layer residual network) and recurrent neural network (long short-term memory network). Area under the curve (AUC) was used to assess myopia and high myopia risk prediction, and mean absolute error (MAE) was used to assess spherical equivalent refraction (SER) prediction. Myopia was defined as SER of –0.5 D or less, and high myopia was defined as –6.0 D or less, using cycloplegic autorefraction.

**Results:**

Among the 3048 children (mean [SD] age, 7.1 [0.4] years; 1716 females [56.3%]) included, the baseline prevalence rate of myopia was 5.71% (174) and high myopia was 0.5% (15). The deep learning model achieved AUC scores of 0.941 (95% CI, 0.936-0.946) for myopia risk prediction and 0.985 (95% CI, 0.982-0.988) for high myopia risk prediction, with an overall MAE of 0.322 D per year for SER prediction. External validation in the Beijing cohort (n = 130; mean [SD] age, 9.9 [3.7] years; 82 males [63.1%]; 128 Han Chinese [98.5%], 2 Man Chu [1.5%]) and the Lhasa cohort (n = 1039; mean [SD] age, 6.8 [0.5] years; 536 males [51.6%]; 29 Han Chinese [2.8%], 1007 Tibetan [96.9%], 3 [0.2%] from other ethnic minority groups) demonstrated maintained cross-ethnicity performance by the model, with an MAE of 0.355 D and 0.261 D per year, respectively.

**Conclusions and Relevance:**

In this cohort study, a deep learning model, using minimal baseline data, provided highly accurate prediction of myopia and high myopia risk. This deep learning approach suggests the utility of large-scale screening and early-intervention efforts in resource-limited settings.

## Key Points

**Question:**

Can deep learning methods accurately predict childhood myopia progression using only fundus images and baseline refraction data?

**Findings:**

In this cohort study of 3048 schoolchildren with 16 211 fundus images, a novel deep learning model achieved high accuracy in predicting myopia and high myopia risk as well as a mean absolute error of 0.322 D per year for refraction.

**Meaning:**

This deep learning approach enables accurate prediction of myopia progression without extensive metadata and may facilitate large-scale screening and early-intervention efforts in resource-limited settings.

## Teaser

This cohort study introduces a deep-learning method for assessing myopia and high myopia risk in schoolchildren in China using fundus images and baseline refraction data.

## Introduction

High myopia is anticipated to affect more than 1 billion individuals by 2050 and significantly increases risk of severe complications, including myopic maculopathy and permanent vision loss.^1^ Given the irreversible progression of high myopia, early intervention remains the most effective preventive strategy,^2,3^ as childhood myopia onset strongly correlates with later development of high myopia.^4^

Three challenges impede effective intervention. First, while pharmacologic interventions can reduce myopic progression by more than 70% in children aged 6 to 13 years,^5^ the lack of predictive ability regarding individual myopic trajectories impedes timely treatment.^6^ Second, experienced ophthalmologists can identify high myopia risk through ocular examination, but this assessment is subjective and requires substantial expertise. Third, myopia prevalence continues to increase globally, with rates exceeding 90% in some Asian countries.^7^ Given the known disparities in myopia prevalence across ethnic groups, prediction models require validation in diverse populations. Machine learning models used in research^8,9,10^ that incorporate multidimensional data show potential utility, but further innovation is warranted to develop efficient scalable methods for accurately predicting myopia progression at early stages.

Fundus imaging provides a noninvasive and cost-effective screening tool that captures subtle changes preceding clinically detectable myopia progression—changes that traditional factors alone such as spherical equivalent refraction, age, and parental myopia cannot identify.^11,12^ Preliminary studies have explored the use of fundus images alongside baseline physiological data to predict myopia risk in children.^13,14^ While fundus images have been successfully used to diagnose severe retinal complications,^15,16,17,18^ current methods have not accurately identified children at high risk within 3 years of myopia progression or quantitatively predicted myopia progression. This study aimed to develop and validate a quantitative method, based on deep learning that combines convolutional neural network (34-layer residual network [ResNet34]^19^) and recurrent neural network (long short-term memory [LSTM] network^20^) and using only fundus images and baseline refraction data, to predict both myopia progression trajectory and high myopia risk in schoolchildren. This deep learning approach provides multiyear risk assessment and personalized quantitative development trajectories using as little as a single fundus photograph and initial diopter readings, positioning it as a crucial tool for widespread screening and clinical decision-making across diverse health care settings.

## Methods

### Design and Setting

This prospective longitudinal school-based cohort study (Anyang Childhood Eye Study [ACES]) was designed to assess myopia progression risk in Chinese children over a 6-year period using deep learning analysis of fundus images and refractive data. ACES was conducted in urban areas of Anyang city in Henan Province, Central China, and adhered to the tenets of the Declaration of Helsinki.^21^ The study protocol for the Anyang Childhood Eye Study has been previously published.^22^ The study was not registered. All parents provided written informed consent for their participating children, and verbal consent was also obtained from each child. The Beijing Tongren Hospital, Capital Medical University Institutional Review Board approved this study. We followed the Strengthening the Reporting of Observational Studies in Epidemiology (STROBE) reporting guideline for the cohort study design and the Transparent Reporting of a Multivariable Prediction Model for Individual Prognosis or Diagnosis (TRIPOD) reporting guideline for the prediction model development and validation.

Details on the methods and sample size calculations have been published elsewhere.^22^ Children enrolled in ACES underwent a comprehensive standardized examination at both baseline and follow-up. Recruitment occurred between February and May 2012, with annual follow-up examinations from February 2013 to May 2018. Data analysis was performed from July 2024 to July 2025. The study design and data analysis framework is provided in eFigure 1 in Supplement 1.

### Participants

Grade 1 students, aged 6 to 9 years, were recruited from 11 randomly selected primary schools in Anyang. Children who had received or were currently undergoing myopia control treatments, who had amblyopia, or who underwent strabismus surgery were excluded. Annual follow-up examinations were conducted at the same schools using identical protocols, yielding a total of 16 211 fundus images. Children who transferred schools were contacted using family contact information to facilitate continued participation when possible. Loss to follow-up and reasons for the loss were documented.

### Variables and Definition of Myopia

Spherical equivalent refraction (SER) was defined as spherical power plus half cylinder from cycloplegic refraction. Cycloplegia was induced following a standardized protocol: after corneal anesthesia with 1 drop of topical anesthetic agent (Alcaine; Alcon Inc), 2 drops of 1% cyclopentolate (Alcon Inc) and 1 drop of tropicamide (Mydrin-P; Santen Pharmaceutical Co) were administered with an interval of 5 minutes between drops. Thirty minutes after tropicamide administration, cycloplegia was considered adequate if the pupillary light reflex was absent or a pupil size of more than 6.0 mm. If these criteria were not met, a third drop of cyclopentolate was administered. Cycloplegic refraction was then measured using 3 repeated measurements by an autorefractor (HRK-7000 A; Huvitz).

Myopia was defined as SER of −0.5 D (diopter) or less, and high myopia was defined as SER of −6.0 D or less. Rapid myopia progression was defined as an increase in the myopic SER greater than 0.75 D per year among children with myopia. Children with SER progression of less than 0.50 D per year had slow myopia progression.

### Image Preprocessing and Deep Learning Model

Fundus images were captured using a nondilated fundus camera (Canon CR-2; Canon). We developed an image preprocessing system standardizing input to 512 × 512 × 3 (width × height × color channels for red, green, and blue) and high-pass filtering enhancement (eFigure 2 in Supplement 1). We used gradient-weighted Class Activation Mapping (Grad-CAM) and guided backpropagation to draw weight heat maps for the deep learning model. These heat maps were used to predict future risk for high myopia and to quantitatively predict the myopic shift. These 2 technologies (Grad-CAM and guided backpropagation) offer different methods of attributing some of the contributions of a multiyear myopia prediction network (MMPN) to the regions in the image.

We developed the MMPN by implementing a model integration strategy (eFigure 3 in Supplement 1). In the MMPN, model 1 belongs to the encoder component, through which features are extracted from the input image sequence using the ResNet34 structure based on the convolutional neural network architecture and are retained in the final convolutional layer. These feature maps are then converted into feature vectors using global average pooling and spliced with the refractive index sequence feature values as the final output of the encoding part, which also serves as the input for the decoding component. Model 2 in the MMPN belongs to the decoding component, which uses the LSTM structure in the recurrent neural network architecture to process temporal information and perform predictions, outputting the future refractive index sequence. For model training, we used Python 1.12.0 (Python Software Foundation) with A5000-24G graphics acceleration. The transformation of the performance of the NPM model, where N represents the input sequence length and M represents the prediction length, is shown in eFigure 7 in Supplement 1. Predictors were selected based on clinical rationale and previous literature. Initial predictors included fundus images and baseline SER. No formal predictor selection procedures were performed before model building, as our approach was designed to use minimal baseline data. Model fairness was assessed through subgroup analyses stratified by sex and baseline myopic status. Performance metrics were reported separately for these groups to identify potential disparities (eFigure 4 in Supplement 1).Outcome assessment was fully objective and did not require subjective interpretation.

### External Validation Datasets

To assess generalizability across different populations, we conducted external validation using 2 independent cohorts. The first cohort was the Lhasa cohort from the Tibet Autonomous Region, which consisted of Han Chinese, Tibetan, and other ethnic minority (including Man Chu, Mongolian, Yao, and Bai) children. This high-altitude population represented distinct environmental and genetic backgrounds. The second cohort was the Beijing cohort, which comprised primarily Han Chinese children who were initially screened from urban schools in Beijing. Participant ethnicity was identified from parental report during enrollment.

Fundus images were captured using the same imaging protocol as that in internal validation, and cycloplegic refraction measurements were obtained following the aforementioned procedures. For both the Lhasa and Beijing external validation cohorts, the definitions for myopia (SER ≤−0.5 D) and high myopia (SER≤−6.0 D) were the same, and the same image preprocessing and model architecture were applied without any retraining or fine-tuning.

### Statistical Analysis

All examination data were entered twice into a database using EpiData software, version 3.1 (EpiData Association), by 2 trained data entry clerks. All analyses were conducted using SPSS software, version 20.0 (SPSS Inc). A 2-sided *P* < .05 was considered statistically significant. We calculated 95% CIs using bootstrap methods with 1000 iterations. Missing fundus images due to poor quality were excluded from analysis. Multiple imputation was not performed because missing data were assumed to be missing completely at random. Subgroup analyses stratified by sex and baseline myopia status were performed to assess the deep learning model’s performance across different demographic groups. Sample size was determined based on requirements for deep learning model development, following the principle of at least 10 events per predictor variable for the binary outcomes and considerations for temporal sequence modeling. With 16 211 fundus images from 3048 participants including 1538 incident myopia cases and 143 incident high myopia cases over 5 years, the sample size was considered adequate for model development and internal validation. For external validation, a minimum of 100 outcome events was targeted to ensure stable performance estimates.

Class imbalance methods were not used. The model was trained on the natural class distribution (baseline myopia: 5.71%; high myopia: 0.49%). Performance was evaluated separately for balanced and imbalanced scenarios.

In performance evaluation, we used area under the (receiver operating characteristic) curve (AUC) for risk prediction and mean absolute error (MAE) for quantitative SER prediction.^23^ When diagnosing myopia in children and predicting myopia progression, we used visual interpretation tools to understand which regions in the fundus image had the greatest implications for the deep learning model.

## Results

**Figure 1.**
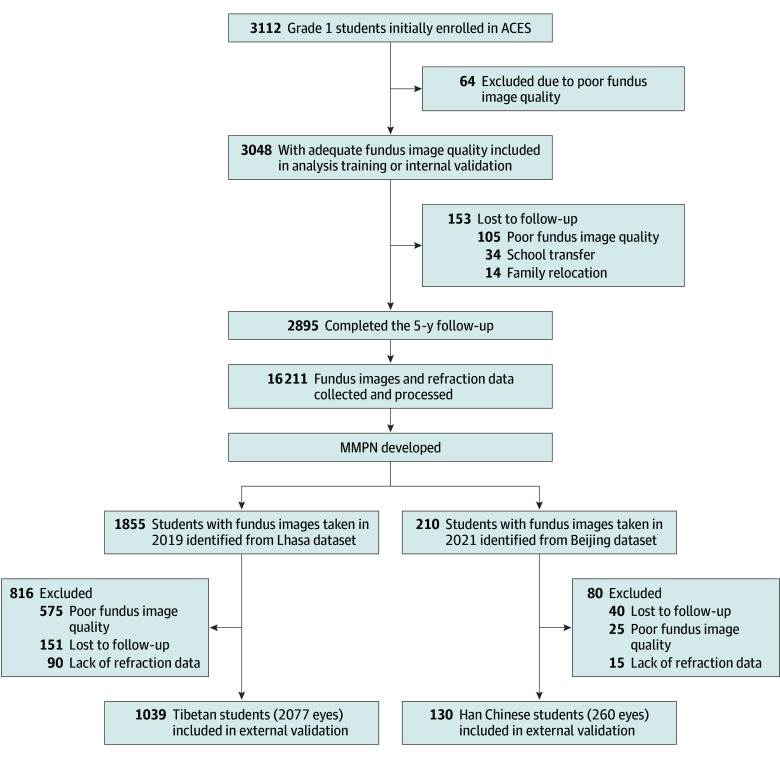
Anyang Childhood Eye Study (ACES) Participant Flowchart Deep learning system combining convolutional (ResNet34-based) and recurrent (LSTM [long short-term memory]) neural networks was used for fundus image sequence analysis training or internal validation and external validation. MMPN indicates Multiyear Myopia Prediction Network.

Of the 3112 grade 1 students initially enrolled in ACES, 3048 (97.9%) met the inclusion criteria and 2895 (95.0%) completed the 5-year follow-up and had adequate fundus image quality available for analysis. At baseline, the 3048 patients had a mean (SD) age of 7.1 (0.4) years and included 1716 females (56.3%) and 1332 males (43.7%). Among 2734 children with ethnicity data available from parental questionnaires, 2723 (99.6%) were identified as Han Chinese and 11 (0.4%) as other ethnicities (6 Hui, 2 Qiang, 1 Man Chu, 1 Tujia, and 1 Yao). Loss to follow-up occurred due to school transfers (n = 34), family relocation (n = 14), and poor fundus image quality (n = 105) (Figure 1). No significant differences in baseline characteristics were observed between participants who completed the study and those lost to follow-up. The baseline myopia prevalence rate was 5.7% (174 of 3048), and the baseline high myopia prevalence rate was 0.5% (15 of 3048). Over the 5-year follow-up period, among 2730 children who were initially nonmyopic, 1538 (56.3%) developed myopia, yielding an incidence rate of 56.3%. Among 2881 children without high myopia at baseline, 143 (5.0%) developed high myopia, representing an incidence rate of 5.0% or 9.9 per 1000 person-years. A total of 16 211 fundus images from 3048 participants were used to develop and internally validate the deep learning model. Demographic and ocular characteristics at baseline are provided in eTable 1 in Supplement 1.

### Internal Validation Performance

**Figure 2.**
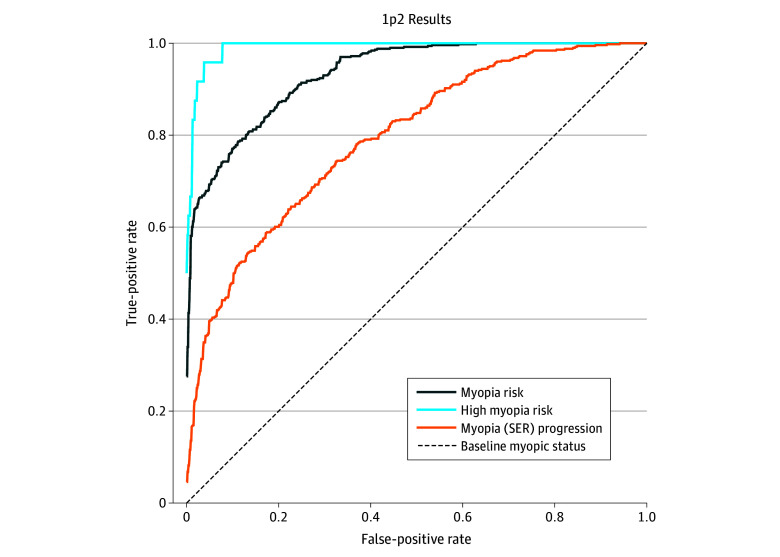
Deep Learning Model Performance for Myopia Risk Assessment and Progression Prediction Area under the curve (AUC) scores exceeded 0.933 for both myopia risk (0.813) and high myopia risk (0.991) in the subsequent year. The notation 1p2 indicates use of year-1 data to assess year-2 future risk. SER indicates spherical equivalent refraction.

The deep learning model achieved AUC scores of 0.941 (95% CI, 0.936-0.946) for myopia risk prediction and 0.985 (95% CI, 0.982-0.988) for high myopia risk prediction, with an overall MAE of 0.322 D per year for SER prediction. Predictive accuracy was 0.870 (95% CI, 0.868-0.972) for myopia risk and 0.979 (95% CI, 0.978-0.980) for high myopia risk. For fundus images only, the AUC score was 0.989 (95% CI, 0.981-0.997). Using single-visit data, the model predicted 3-year myopia risk (AUC, 0.888; 95% CI, 0.865-0.911) with an MAE of 0.235 D per year. Details of the algorithm performance validation are presented in the Table. The MAEs per year in predicting myopic SER over the next 1 to 5 years using only 1 year of data (ie, year-1 data used to assess year-1 to year-5 risk or outcome) were 0.369 D per year for 1-year predictions, 0.554 D per year for 2-year predictions, 0.704 D per year for 3-year predictions, 0.906 D per year for 4-year predictions, and 1.098 D per year for 5-year predictions. Model performance in myopia risk assessment and progression prediction is provided in Figure 2. Additional NPM model results are shown in eFigures 5 and 6 in Supplement 1.

**Table.** Deep Learning Model Performance for Myopia and High Myopia Risk Prediction

Model^a^	SER quantitative prediction		Risk prediction measure (95% CI)^b^						
	MAE per y, D	*R* ^2^	Trained classifier^c^				Threshold classifier^c^		
			Accuracy	Sensitivity	Specificity	AUC	Accuracy	Sensitivity	Specificity
1p1									
Myopia	0.369	0.904	0.913 (0.909-0.917)	0.917 (0.913-0.920)	0.906 (0.902-0.910)	0.973 (0.965-0.981)	0.924 (0.921-0.928)	0.918 (0.915-0.922)	0.935 (0.932-0.938)
High myopia			0.992 (0.991-0.993)	0.992 (0.991-0.993)	1.000 (1.000-1.000)	0.999 (0.997-1.00)	0.984 (0.983-0.986)	0.995 (0.994-0.996)	0.890 (0.886-0.894)
1p2									
Myopia	0.554	0.971	0.866 (0.861-0.872)	0.869 (0.864-0.874)	0.863 (0.858-0.868)	0.933 (0.918-0.948)	0.883 (0.878-0.888)	0.873 (0.868-0.878)	0.896 (0.892-0.901)
High myopia			0.986 (0.985-0.988)	0.986 (0.985-0.988)	1.000 (1.000-1.000)	0.991 (0.985-0.997)	0.995 (0.994-0.996)	0.998 (0.997-0.998)	0.903 (0.898-0.907)
1p3									
Myopia	0.704	0.709	0.787 (0.779-0.795)	0.722 (0.714-0.731)	0.843 (0.836-0.849)	0.888 (0.865-0.911)	0.823 (0.816-0.830)	0.817 (0.810-0.825)	0.831 (0.823-0.838)
High myopia			0.984 (0.982-0.987)	0.986 (0.983-0.988)	0.875 (0.869-0.881)	0.989 (0.981-0.997)	0.980 (0.977-0.983)	0.992 (0.990-0.993)	0.651 (0.642-0.659)
1p4									
Myopia	0.906	0.660	0.758 (0.748-0.768)	0.723 (0.712-0.734)	0.791 (0.781-0.801)	0.858 (0.825-0.891)	0.798 (0.788-0.808)	0.740 (0.730-0.751)	0.841 (0.832-0.850)
High myopia			0.973 (0.969-0.977)	0.974 (0.970-0.978)	0.889 (0.881-0.897)	0.928 (0.904-0.952)	0.982 (0.979-0.985)	0.990 (0.988-0.993)	0.218 (0.208-0.228)
1p5									
Myopia	1.098	0.549	0.727 (0.711-0.743)	0.688 (0.672-0.705)	0.755 (0.739-0.770)	0.782 (0.724-0.840)	0.765 (0.750-0.780)	0.687 (0.670-0.703)	0.808 (0.794-0.822)
High myopia			0.957 (0.950-0.964)	0.957 (0.950-0.964)	1.000 (1.000-1.000)	0.911 (0.871-0.951)	0.991 (0.988-0.995)	1.000 (1.000-1.000)	0.000 (0.000-0.000)
Mean per y^d^									
Myopia	0.322	0.860	0.870 (0.868-0.972)	0.852 (0.850-0.854)	0.889 (0.887-0.891)	0.941 (0.936-0.946)	0.874 (0.872-0.876)	0.853 (0.851-0.855)	0.896 (0.894-0.898)
High myopia			0.979 (0.978-0.980)	0.979 (0.979-0.980)	0.962 (0.961-0.963)	0.985 (0.982-0.988)	0.986 (0.986-0.987)	0.994 (0.993-0.994)	0.700 (0.697-0.702)

Abbreviations: AUC, area under the (receiver operating characteristic) curve; D, diopter; MAE, mean absolute error; SER, spherical equivalent refraction.

^a^ Model configurations are stratified by prediction sequence length, where 1p1 indicates year-1 baseline data predicting year-1 outcomes or future risk, 1p2 indicates year-1 baseline data predicting year-2 outcomes, and so on.

^b^ The table shows the performance of the classification task for myopia risk prediction and the regression task for quantitative prediction of refractive error development.

^c^ The trained classifier refers to the use of deep learning techniques to classify the final features of the deep learning model, while the threshold classifier refers to the use of quantitative prediction of myopia progression to directly classify future myopia risk using thresholds.

^d^ The final mean was obtained by considering the amount of data from the different models and the weighted mean of the forecast years. Further internal validation analysis is provided in eTable 1 in Supplement 1.

### Model Interpretability

**Figure 3.**
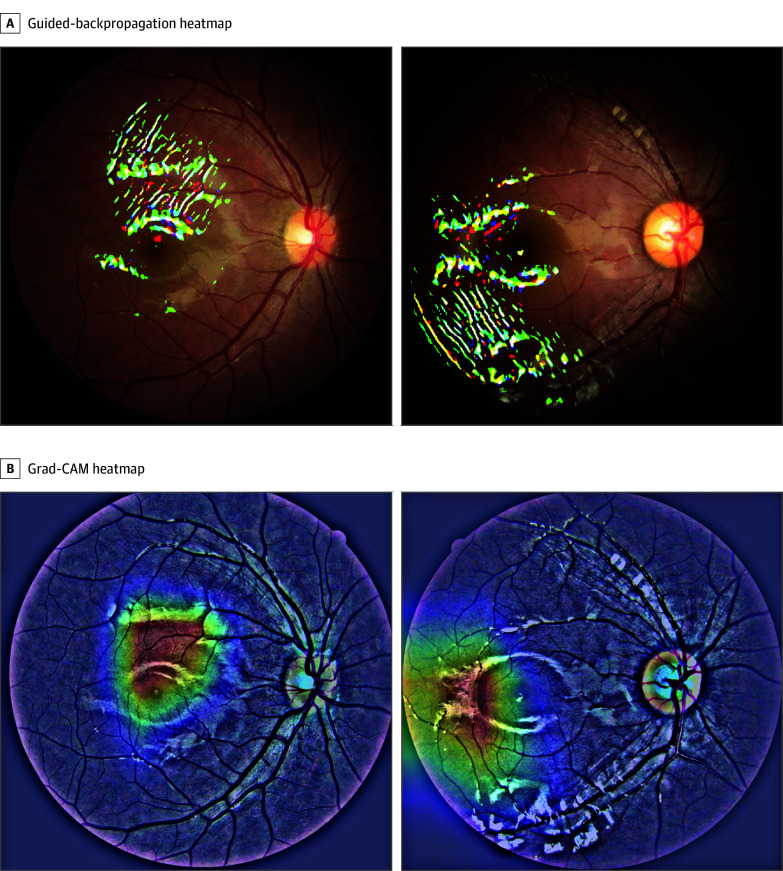
Fundus Image Preprocessing and Model Attention Visualization Using Gradient-Weighted Class Activation Mapping (Grad-CAM) and Guided Backpropagation Four typical images were selected to generate the heat map of rapid progression (myopia shift of more than 0.75 D per year).

Grad-CAM visualization and guided backpropagation heat maps revealed that the deep learning model’s focus on temporal superior and inferior retinal areas, posterior pole changes, and optic disc features aligned with established clinical markers of myopia progression and areas of future high myopia complications (Figure 3). The model’s ability to quantitatively assess these subtle morphological changes provides an objective measure that complements refractive data, enabling more precise risk stratification for children with similar baseline refractive characteristics but different progression trajectories. A comparison between the deep learning model and traditional methods is shown in eTable 2 in Supplement 1.

### Subgroup Analysis

**Figure 4.**
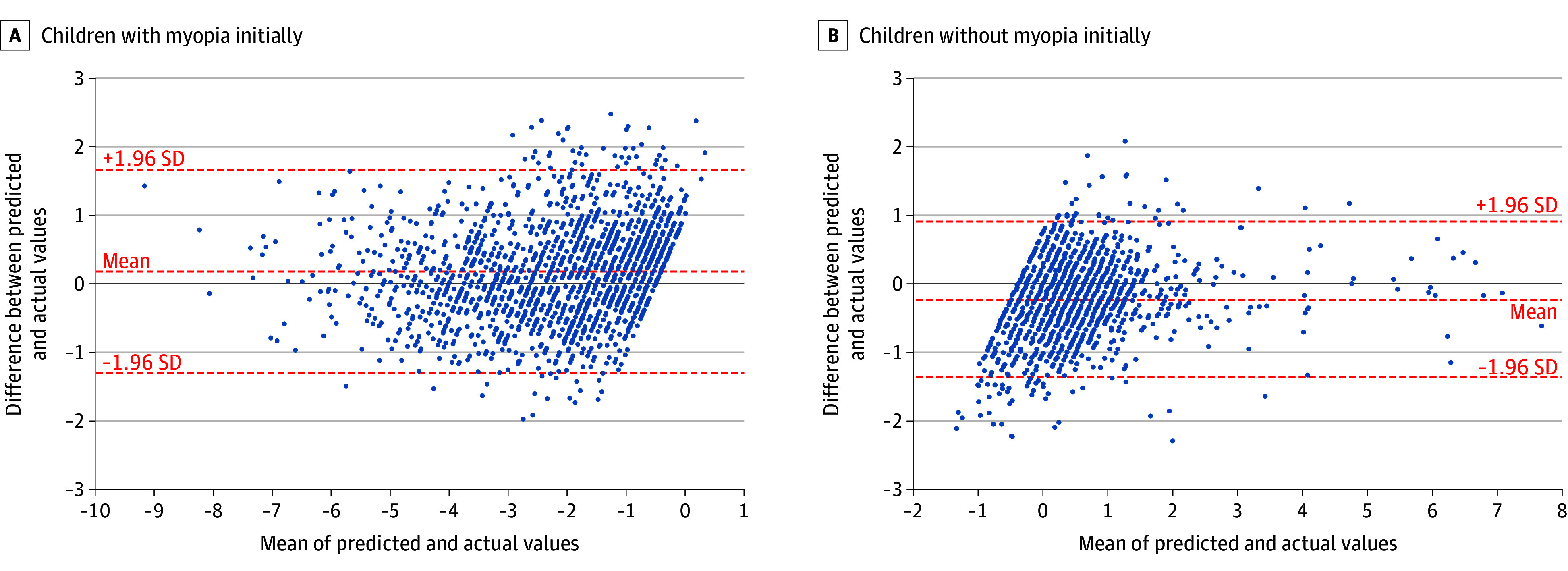
Bland-Altman Analysis of Deep Learning Model Performance by Baseline Myopic Status The prediction accuracy had a mean absolute error (MAE) of 0.58 D per year for children with myopia initially (at baseline) and an MAE of 0.44 D per year for children without myopia initially. Dashed red lines indicate 95% limits of agreement (plus or minus 1.96 SD of the mean), demonstrating superior model performance in nonmyopic children with tighter agreement bounds.

Subgroup analysis revealed significant model performance disparities by baseline myopic status (Figure 4). For children without myopia initially, the MAE was 0.44 D per year, compared with 0.58 D per year for children with myopia initially. Sex-based analysis showed minimal differences (less than 5%) in MAE between male and female participants, confirming that sex was not associated with model performance and baseline myopic status had a primary role in prediction accuracy (eFigure 4 in Supplement 1).

### External Validation Performance

The deep learning model demonstrated robust performance across both external validation cohorts, which had distinct ethnic compositions. The Lhasa cohort comprised 1039 children (mean [SD] age, 6.8 [0.5] years; 503 females [48.4%], 536 males [51.6%]) with 2077 eyes. Among these children, 29 (2.8%) had Han Chinese; 1007 (96.9%) had Tibetan; and 3 (0.2%) had other (Man Chu, Mongolian, Yao, and Bai) ethnicities. Despite the predominantly Tibetan ethnic composition and high-altitude environmental conditions of this cohort, the model maintained strong predictive performance. For the 2-year myopia progression prediction, the model achieved an MAE of 0.522 D per year—equivalent to an MAE of 0.261 D per year—demonstrating maintained predictive accuracy across the cohort’s ethnic groups. Myopia risk assessment yielded an AUC score of 0.913 (95% CI, 0.901-0.925), with accuracy of 0.893 (95% CI, 0.893-0.896). High myopia risk assessment yielded an AUC score of 0.967 (95% CI, 0.959-0.975), with accuracy of 0.998 (95% CI, 0.998-0.999). The model’s consistent performance across the ethnically distinct Lhasa cohort validates the model’s ability to generalize beyond Han Chinese populations.

In the Beijing cohort, 210 children were initially screened. After exclusions, 130 children (mean [SD] age, 9.9 [3.7] years; 48 females [36.9%], 82 males [63.1%]; 128 Han Chinese [98.5%], 2 Man Chu [1.5%]) with 260 eyes were included in the analysis. For the 1-year myopia progression prediction using baseline data, the model achieved an MAE of 0.355 D per year, which was comparable to the internal validation performance (MAE of 0.369 D per year for 1-year predictions). High myopia risk prediction demonstrated perfect discrimination, with an AUC score of 1.00 (95% CI, 1.00-1.00) (eFigure 8 in Supplement 1).

## Discussion

In this study, we introduced a novel deep learning approach for predicting myopia progression in 3048 children using 16 211 fundus images and baseline refractive data over 6 years. This deep learning model achieved accuracy of 0.870 for myopia risk with AUC score of 0.941 and accuracy of 0.979 for high myopia risk predictions with AUC score of 0.985, and an MAE of 0.322 D per year for SER prediction. While this method can provide predictions based on a single clinical visit, effective myopia management still requires regular follow-up assessments.

External validation demonstrated maintained performance across ethnically distinct populations. The Lhasa cohort, consisting predominantly of Tibetan participants, achieved an MAE of 0.261 D per year and an AUC score of 0.967 for high myopia prediction, providing evidence for cross-ethnicity applicability. Generalizability to other major populations (African, European, and South Asian) was not established, and the findings require validation in more ethnically diverse cohorts.

Previous predictive methods by Zadnik et al^24^ and Lin et al^10^ achieved comparable accuracy (AUC scores from 0.87 to 0.976) but required substantial multivisit data collection, limiting their utility in resource-constrained settings. Foo et al^13^ combined fundus images with baseline refractive data and achieved an AUC score of 0.97 over 5 years. Our approach differs by extracting more comprehensive information from fundus images while requiring minimal clinical data. A direct comparison of the deep learning model to state-of-the-art methods reveals significant methodological and performance distinctions (eFigure 9 in Supplement 1). While Foo et al^13^ focused on binary high myopia classification over 5 years in 998 Singaporean children (aged 6-12 years) using 8277 images, we addressed both quantitative myopia progression prediction and high myopia risk assessment over 6 years in a substantially larger cohort (3048 Chinese children aged 6-13 years; 16 211 images). Our model demonstrated superior performance (image-only AUC, 0.989 vs 0.93; overall AUC, 0.985 vs 0.97) and unique quantitative prediction capabilities (MAE, 0.322 D per year) with single-visit data, whereas other methods in previous studies required progression data for optimal performance.

The deep learning model we developed and validated advances the analysis of fundus images through a sophisticated preprocessing system that accentuates physiological characteristics relevant to myopia progression. Unlike diseases with distinct retinal features,^25,26^ normal myopia lacks readily discernible characteristics, complicating prediction model development. Our approach offers key advantages, including data efficiency (single-visit requirements), predictive versatility (quantitative progression trajectories), and implementation flexibility in resource-limited settings. Through Grad-CAM visualization, this model identifies potential biomarkers concentrated in the optic disc, macular area, and temporal retinal regions, consistent with areas of future high myopia complications, including maculopathy, dome-shaped macula, and posterior staphyloma.^27,28,29^

However, our findings reveal fundamental challenges that align with those discussed in extensive literature on myopia prediction. The higher prediction errors in children who were myopic at baseline (31.8% increase in MAE) reflect the inherent complexity of myopia progression documented in landmark studies. The CLEERE (Collaborative Longitudinal Evaluation of Ethnicity and Refractive Error) study^30,31^ demonstrated that, while early refractive error may indicate myopia onset, progression patterns remain highly unpredictable, with progression history providing limited clinical utility for treatment decisions. These findings highlight architectural considerations for temporal modeling in myopia research. While LSTM networks excel at identifying patterns in sequential data, myopia progression after onset exhibits high variability and divergent patterns that challenge traditional time-series approaches. Our results suggest that temporal dependencies in established myopia cases may be too weak or inconsistent to support reliable prediction using sequence-based architectures, consistent with CLEERE findings and other longitudinal research demonstrating the inherent unpredictability of myopia progression patterns.

The multifactorial nature of myopia progression presents unique challenges for predictive modeling. Failure analysis (eFigure 10 in Supplement 1) reveals distinct failure modes that provide insights into model limitations. It demonstrates 3 primary patterns: (1) peripheral vascular pattern misattribution, where model attention focuses on retinal vessel configurations that may not directly correlate with refractive progression; (2) detection threshold limitations, where early morphological changes remain below conventional imaging detection thresholds; and (3) temporal prediction challenges in cases with irregular progression patterns that deviate from typical developmental trajectories. These observations highlight the fundamental challenge of using fundus imaging alone to capture the multifactorial nature of myopia progression, where environmental, lifestyle, and genetic factors may manifest through subtle changes that are not readily apparent in standard fundus photography.

Despite these challenges, the deep learning model demonstrated that clinically meaningful predictions can be achieved using minimal baseline data, particularly for identifying children at risk before myopia onset. Findings of this study highlight the need for further research to develop more comprehensive modeling approaches that can better integrate multiple influential factors, potentially through multimodal data fusion and architectures that are specifically optimized for the unique characteristics of myopia progression in established cases. Early identification of at-risk children could reduce the economic burden and psychological consequences associated with advanced myopia.^32^ Myopia substantially impacts workforce productivity,^33^ with East Asia alone experiencing productivity losses exceeding $140 billion,^34^ and is associated with anxiety disorders and social challenges such as school bullying.^35^ In myopia screening for children and adolescents, fundus photography could be added to visual acuity and refraction examinations to enhance the diagnostic predictive value of screening.^36,37^ For clinical implementation, input data quality should be assessed prior to model application. Fundus images should be evaluated for adequate illumination, focus, and field of view. Images with significant media opacity, motion artifact, or incomplete macular visualization should be flagged for repeat acquisition or excluded from automated analysis. The preprocessing pipeline includes automated quality assessment that rejects images below minimum quality thresholds. When cycloplegic refraction is unavailable, noncycloplegic measurements may be used with the understanding that prediction accuracy may be reduced, particularly in children with hyperopia.

### Limitations

The study has several limitations. First, we were unable to establish the deep learning model’s generalizability performance across diverse populations. While external validation demonstrated its robust performance in Han Chinese and Tibetan populations, the applicability of this finding to other ethnic groups remains unestablished. Mitigating these biases would be possible by fine-tuning the model and using local datasets for model retraining and adjustments in future applications. To enhance the model’s effectiveness, future work should incorporate data encompassing a broader spectrum of regions and races or ethnicities. Second, the underrepresentation of high myopia cases in the dataset may compromise the model’s sensitivity to this condition. Third, the predominance of children with stable myopia progression may introduce systematic bias against children who are exhibiting rapid progression, although this bias reflects general population characteristics.

## Conclusions

This 6-year longitudinal cohort study developed and validated a deep learning model to predict childhood myopia progression using fundus images and baseline refraction data. The results demonstrated this model’s excellent predictive accuracy for myopia and high myopia risk. However, when applied to predict future progression rates in children with myopia at baseline, the model’s accuracy decreased over time, likely due to myopia being affected by multiple environmental and genetic factors. This limitation should be considered in large-scale applications and implementation in different demographic subgroups. Overall, the model may be potentially used for large-scale screening and early- intervention efforts in resource-limited settings.

## Notes

Supplement 1. **eTable 1.** Baseline Characteristics and Data Summary by Model Configuration **eTable 2.** Performance Comparison Between Deep Learning Model and Traditional Methods **eFigure 1.** Study Design and Data Analysis Framework for Deep Learning-Based Childhood Myopia Progression Prediction **eFigure 2.** Image Preprocessing and Enhancement System **eFigure 3.** Multi-Year Myopia Prediction Network (MMPN) Model Architecture **eFigure 4.** Results of Sex-based Subgroup Analyses **eFigure 5.** Results of the Risk Prediction for Myopia and High Myopia **eFigure 6.** Quantitative Prediction of Future Myopia Development Results **eFigure 7.** Overall Performance Matrix of the nPm Model **eFigure 8.** External Validation Performance of Deep Learning Model Across Diverse Populations **eFigure 9.** Performance Comparison Across Myopia Prediction Studies **eFigure 10.** Model Failure Case Analysis and Attention Visualization for Understanding Prediction Limitations

Supplement 2.
Data Sharing Statement
