# Supplementary material for: Deep Learning Prediction of Childhood Myopia Progression Using Fundus Image and Refraction Data
Source: JAMA Netw Open. 2026 Jan 26;9(1):e2553543. doi: 10.1001/jamanetworkopen.2025.53543 (PMC12836131; doi:10.1001/jamanetworkopen.2025.53543)
Supplement: Supplement 1. — eTable 1. Baseline Characteristics and Data Summary by Model Configuration eTable 2. Performance Comparison Between Deep Learning Model and Traditional Methods eFigure 1. Study Design and Data Analysis Framework for Deep Learning-Based Childhood Myopia Progression Prediction eFigure 2. Image Preprocessing and Enhancement System eFigure 3. Multi-Year Myopia Prediction Network (MMPN) Model Architecture eFigure 4. Results of Sex-based Subgroup Analyses eFigure 5. Results of the Risk Prediction for Myopia and High Myopia eFigure 6. Quantitative Prediction of Future Myopia Development Results eFigure 7. Overall Performance Matrix of the nPm Model eFigure 8. External Validation Performance of Deep Learning Model Across Diverse Populations eFigure 9. Performance Comparison Across Myopia Prediction Studies eFigure 10. Model Failure Case Analysis and Attention Visualization for Understanding Prediction Limitations [file jamanetwopen-e2553543-s001.pdf]

Supplemental Online Content

Kang M-T, Hu Y, Wang N, et al. Deep learning prediction of childhood myopia progression using fundus image and refraction data. *JAMA Netw Open*. 2026;9(1):e2553543. doi:10.1001/jamanetworkopen.2025.53543

eTable 1. Baseline Characteristics and Data Summary by Model Configuration
eTable 2. Performance Comparison Between Deep Learning Model and Traditional Methods
eFigure 1. Study Design and Data Analysis Framework for Deep Learning-Based Childhood Myopia Progression Prediction
eFigure 2. Image Preprocessing and Enhancement System
eFigure 3. Multi-Year Myopia Prediction Network (MMPN) Model Architecture
eFigure 4. Results of Sex-based Subgroup Analyses
eFigure 5. Results of the Risk Prediction for Myopia and High Myopia
eFigure 6. Quantitative Prediction of Future Myopia Development Results
eFigure 7. Overall Performance Matrix of the nPm Model
eFigure 8. External Validation Performance of Deep Learning Model Across Diverse Populations
eFigure 9. Performance Comparison Across Myopia Prediction Studies
eFigure 10. Model Failure Case Analysis and Attention Visualization for Understanding Prediction Limitations

This supplemental material has been provided by the authors to give readers additional information about their work.

© 2026 Kang M-T et al. *JAMA Network Open*

eTable1. Baseline Characteristics and Data Summary by Model Configuration

		Model	N	Age (y)	Sex (M%)	DS (D)	DC (D)	Axis (°)	CT (μm)	AL (mm)	SER(D)	Myopia	High myopia
Baseline			3048	7.2	43.74%	1.212	-0.506	99.82	539.96	22.71	0.964	5.71%	0.49%
After 5 years			2895	12.2	43.71%	-1.07	-0.539	103.84	546.65	24.17	-1.356	58.83%	5.42%
The situation of predictive models for sequences of different lengths	Training Set	1p1	7700	9.1	43.13%	0.36	-0.465	90.59	543.47	23.31	0.129	36.90%	1.15%
		1p2	5531	8.6	43.75%	0.633	-0.454	90.48	542.58	23.14	0.402	45.58%	2.18%
		1p3	3538	8.0	43.47%	0.921	-0.459	90.67	542.22	23.02	0.699	53.14%	2.32%
		1p4	2192	7.6	43.20%	1.09	-0.471	90.56	541.24	22.87	0.86	54.24%	4.39%
		1p5	1000	7.2	43.90%	1.271	-0.498	93.99	538.93	22.69	1.023	57.30%	5.01%
		2p1	4745	9.7	43.98%	0.13	-0.458	87.4	544.55	23.5	-0.095	46.32%	2.58%
		2p2	3000	9.1	43.40%	0.425	-0.441	87.88	544.17	23.35	0.214	47.63%	3.08%
		2p3	1696	8.6	44.75%	0.679	-0.444	86.89	543.53	23.24	0.477	58.02%	3.87%
		2p4	700	8.1	43.29%	0.931	-0.44	83.97	543.55	23.22	0.717	58.00%	6.95%
		3p1	2850	10.1	43.02%	-0.066	-0.46	89.6	545.11	23.61	-0.294	50.77%	2.17%
		3p2	1600	9.6	43.63%	0.182	-0.447	89.98	544.82	23.45	-0.04	58.00%	4.58%
		3p3	640	9.1	43.44%	0.487	-0.443	90.06	543.48	23.31	0.288	59.84%	5.65%
		4p1	1500	10.6	43.80%	-0.337	-0.457	89.89	545.74	23.78	-0.557	54.67%	4.67%
		4p2	630	10.1	43.81%	-0.041	-0.436	88.3	543.53	23.59	-0.265	61.75%	5.59%
		5p1	600	11.1	43.33%	-0.618	-0.49	91.44	544.48	23.9	-0.832	58.17%	3.98%
	Validation Set	1p1	1597	9.1	46.65%	0.356	-0.478	89.99	543.5	23.29	0.107	36.88%	1.13%
		1p2	1100	8.6	43.72%	0.647	-0.466	90.42	543.69	23.2	0.411	45.55%	2.18%
		1p3	700	8.0	41.43%	0.854	-0.463	90.92	540.37	22.98	0.62	53.00%	2.43%
		1p4	475	7.6	46.32%	1.116	-0.449	92.99	539.43	22.95	0.888	54.11%	4.21%
		1p5	187	7.2	41.18%	1.215	-0.454	107.96	538.42	22.69	0.990	57.22%	4.81%
		2p1	1000	9.7	39.50%	0.113	-0.45	92.39	545.5	23.45	-0.124	46.40%	2.60%
		2p2	619	9.1	46.37%	0.485	-0.431	89.34	544.77	23.28	0.277	47.50%	2.91%
		2p3	400	8.6	40.75%	0.814	-0.435	86.52	546.84	23.2	0.575	58.00%	3.75%
		2p4	141	8.2	43.97%	0.96	-0.455	75.61	543.58	22.99	0.732	57.45%	7.09%
		3p1	563	10.1	53.46%	-0.057	-0.433	93.78	542.77	23.57	-0.254	50.62%	2.13%
		3p2	393	9.6	41.98%	0.294	-0.391	86.43	541.86	23.43	0.122	58.27%	4.58%
		3p3	151	9.1	41.06%	0.51	-0.49	83.4	542.21	23.24	0.264	59.60%	5.96%
		4p1	385	10.7	42.86%	-0.318	-0.492	94.23	542.63	23.71	-0.539	54.81%	4.94%
		4p2	129	10.1	41.86%	-0.293	-0.414	90.49	548.77	23.7	-0.495	61.24%	5.43%
		5p1	116	11.2	46.55%	-0.373	-0.486	84	42.1	23.89	-0.534	57.76%	4.31%

Notes: Data are presented as means for continuous variables and percentages for categorical variables. N, number of pictures; M, male; DS, diopter of spherical power; DC, diopter of cylindrical power; CT, corneal thickness; AL, axial length; SER, spherical equivalent refraction; NpM, models that process n years (1-5) of fundus image sequences and corresponding optometric data to predict future myopia risk and refractive errors for m subsequent years (1-5). Model configurations are stratified by prediction sequence length, where "1p1" indicates 1-year baseline data predicting 1-year outcomes, "2p3" indicates 2-year baseline data predicting 3-year outcomes, etc. Due to incomplete longitudinal data across all participants, we partitioned the dataset into training and validation sets using a 5:1 ratio while maintaining consistent demographic and clinical feature proportions across both sets. Sample sizes vary across configurations due to different longitudinal data availability requirements for each prediction scenario.

© 2026 Kang M-T et al. *JAMA Network Open*

eTable 2. Performance Comparison Between Deep Learning Model and Traditional Methods

	Mean absolute error/D	Accuracy	Specificity	Sensitivity	AUC
Our method	0.32	87.0%	85.2%	88.9%	0.94
Logistic regression					
Baseline SER	0.85	74.9%	83.2%	63.0%	0.82
Anterior chamber depth	1.25	60.0%	73.1%	73.2%	0.64
Axial length/ corneal radius of curvature	0.77	66.9%	74.5%	56.3%	0.72
Six variables*	0.63	79.1%	82.5%	74.8%	0.84
Random forest（Six variables*）	0.75	80.0%	81.1%	81.2%	0.84

*Uncorrected distance visual acuity, baseline SER, axial length, corneal radius of curvature, gender, parental myopia.

Notes: SER, spherical equivalent refraction; D, diopter; AUC, area under the ROC Curves

© 2026 Kang M-T et al. *JAMA Network Open*

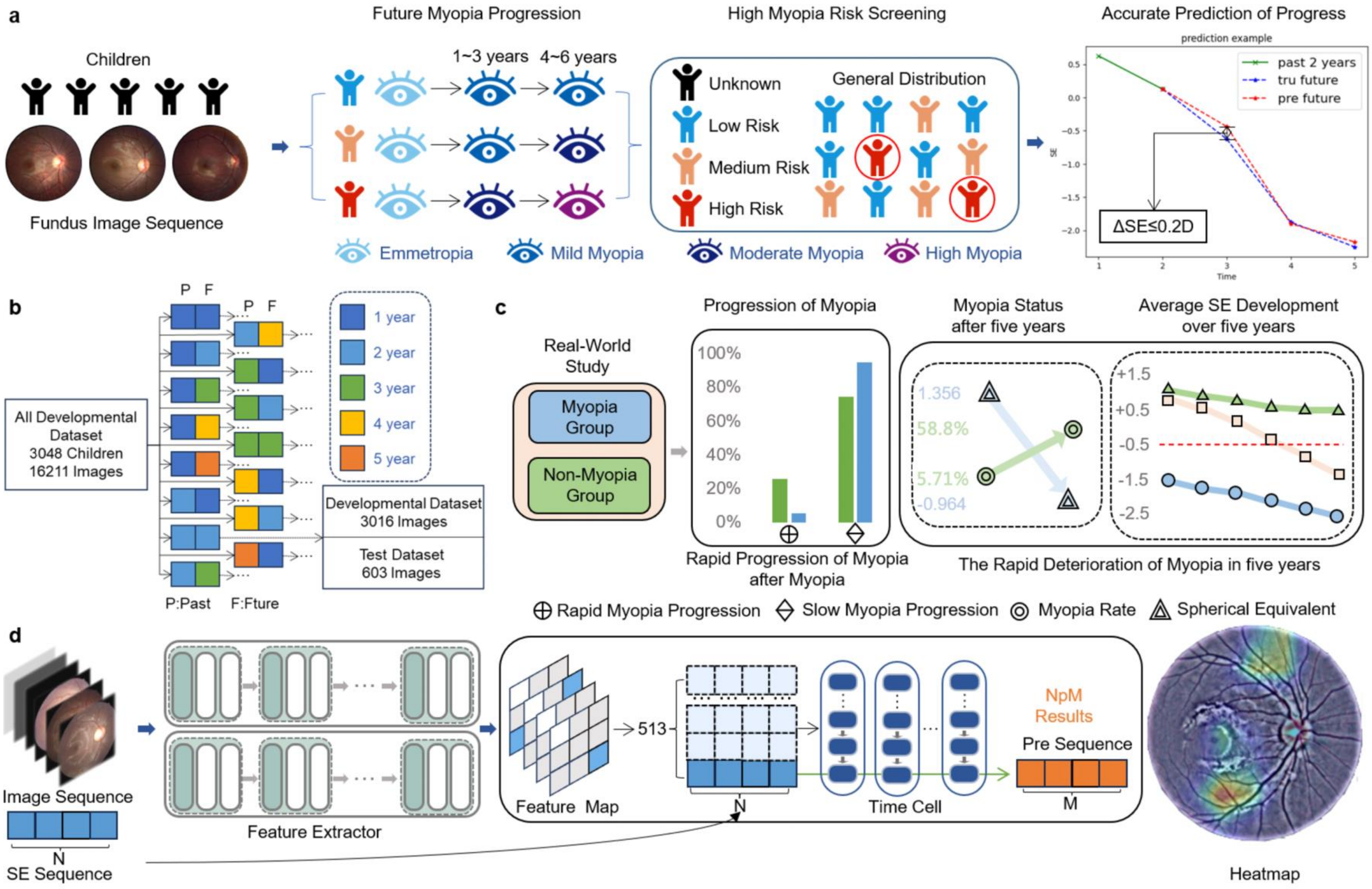

eFigure 1. Study Design and Data Analysis Framework for Deep Learning-Based Childhood Myopia Progression Prediction. **(a)** System schematic for screening high-risk children and quantitative myopia progression prediction. **(b)** Data segmentation methodology for 15 prediction scenarios based on varying baseline and follow-up periods. **(c)** Population characteristics and myopia development patterns in originally myopic versus non-myopic children over five years. **(d)** Deep learning model architecture combining ResNet34 and LSTM networks for fundus image sequence analysis.

© 2026 Kang M-T et al. *JAMA Network Open*

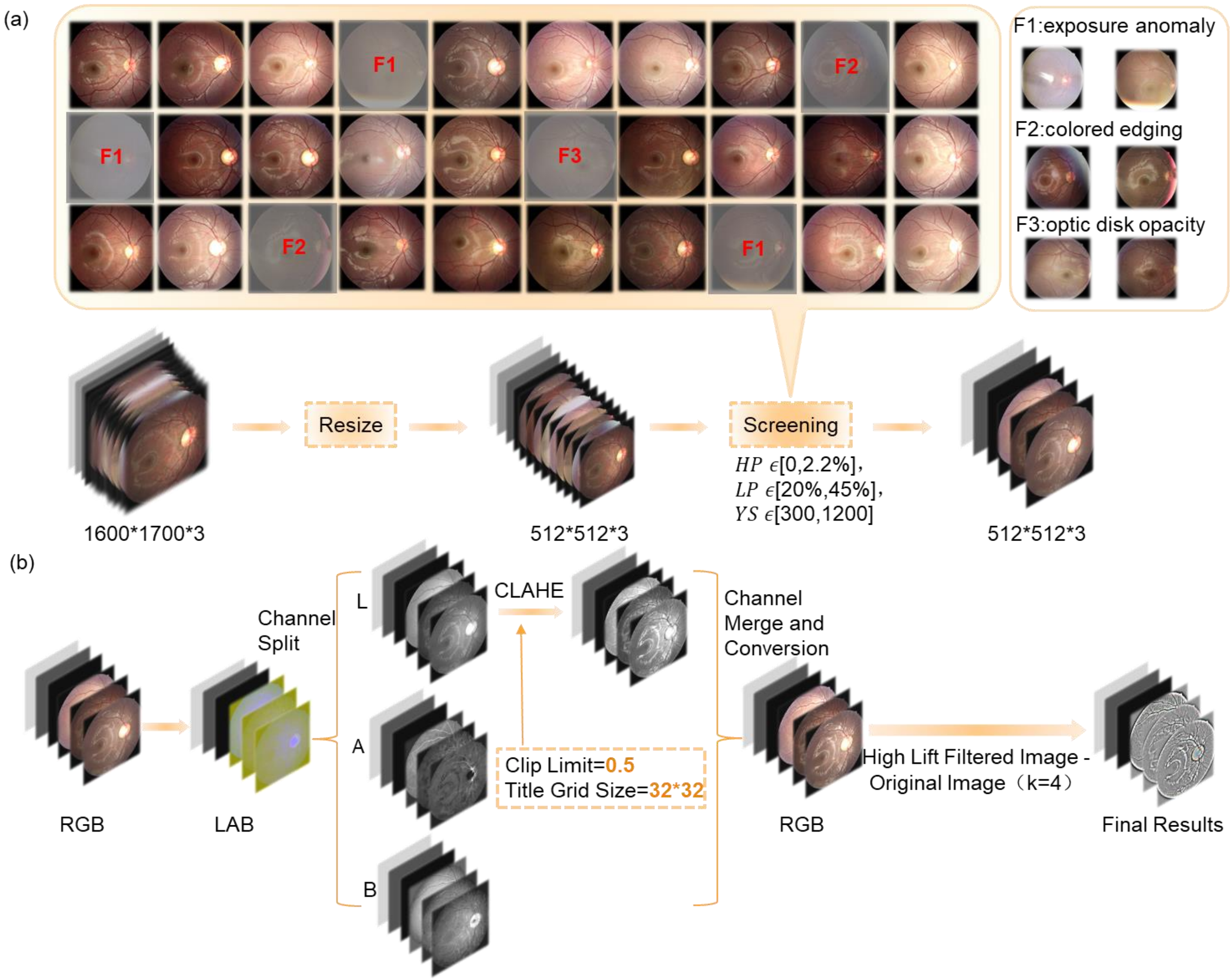

eFigure 2. Image Preprocessing and Enhancement System. Flowchart of the fundus image preprocessing pipeline. The system performs initial cropping and scaling, followed by quality filtering using three manually designed features: HP (high brightness pixel proportion), LP (low brightness pixel proportion), and YS (red channel minus blue channel gray sum). Images are classified into F1, F2, F3 categories for quality control. CLAHE enhancement is applied to the L-channel, followed by high-pass filtering and subtraction operations to enhance key physiological features. Additional augmentation operations (rotation, normalization) are performed during model training.

© 2026 Kang M-T et al. *JAMA Network Open*

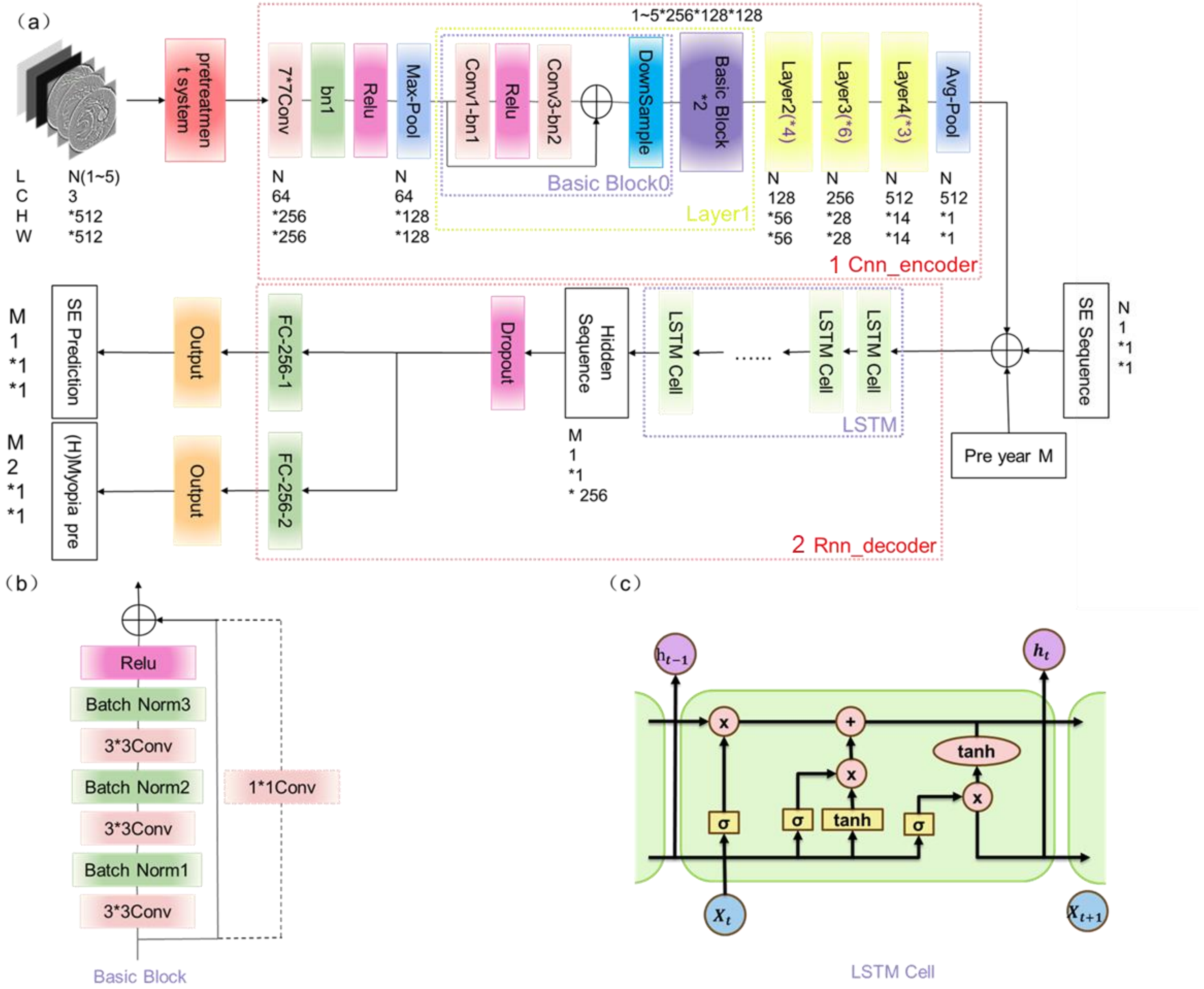

eFigure 3. Multi-Year Myopia Prediction Network (MMPN) Model Architecture. Detailed architecture of the deep learning model showing the integration of convolutional and recurrent neural networks. The model uses ResNet structure for fundus image feature extraction and pooling, fuses extracted features with spherical equivalent refraction (SE) sequences, employs LSTM structure for temporal feature analysis to capture hidden myopia development patterns, and utilizes fully connected layers for final classification or regression tasks.

© 2026 Kang M-T et al. *JAMA Network Open*

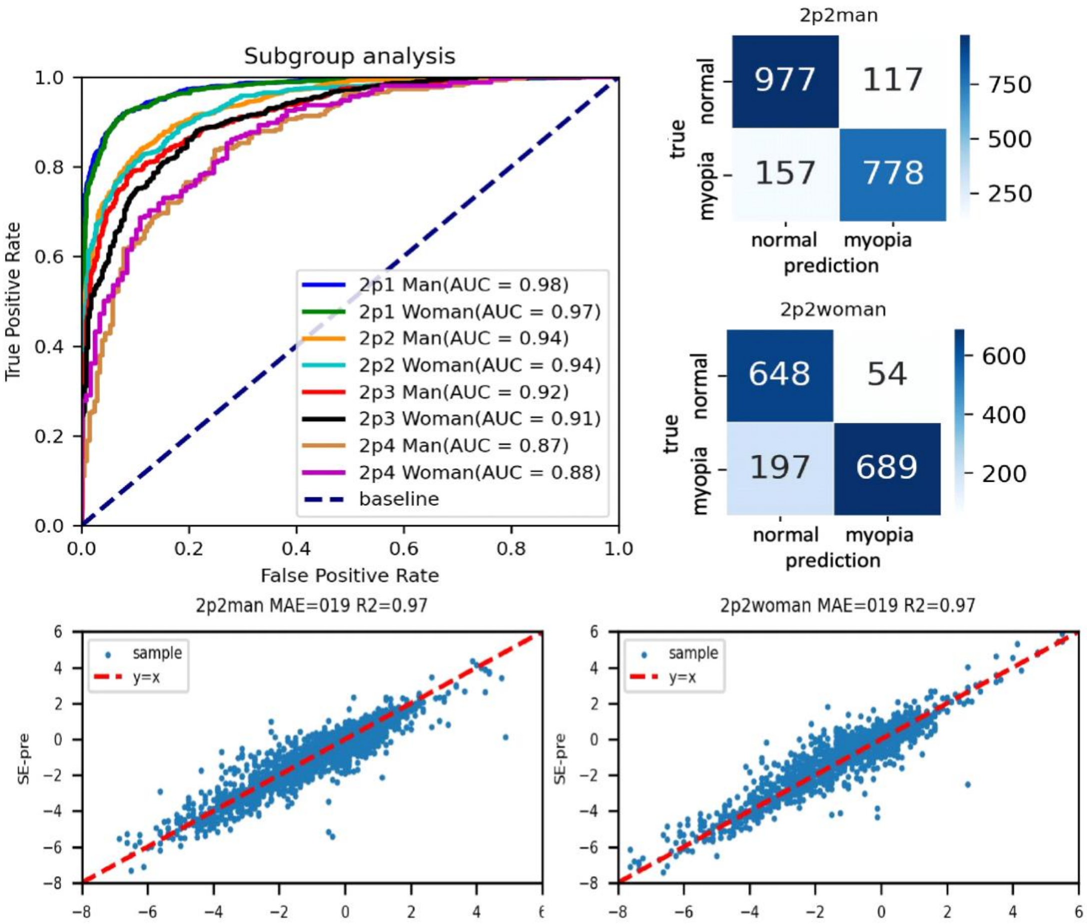

eFigure 4. Results of Sex-based Subgroup Analyses. Performance evaluation across male and female cohorts showing ROC curves, confusion matrices, scatter plots, and regression metrics (MAE, R^2) for various prediction sequences. Results demonstrate consistent model performance across sex groups with minimal differences (<5% MAE variation).

© 2026 Kang M-T et al. *JAMA Network Open*

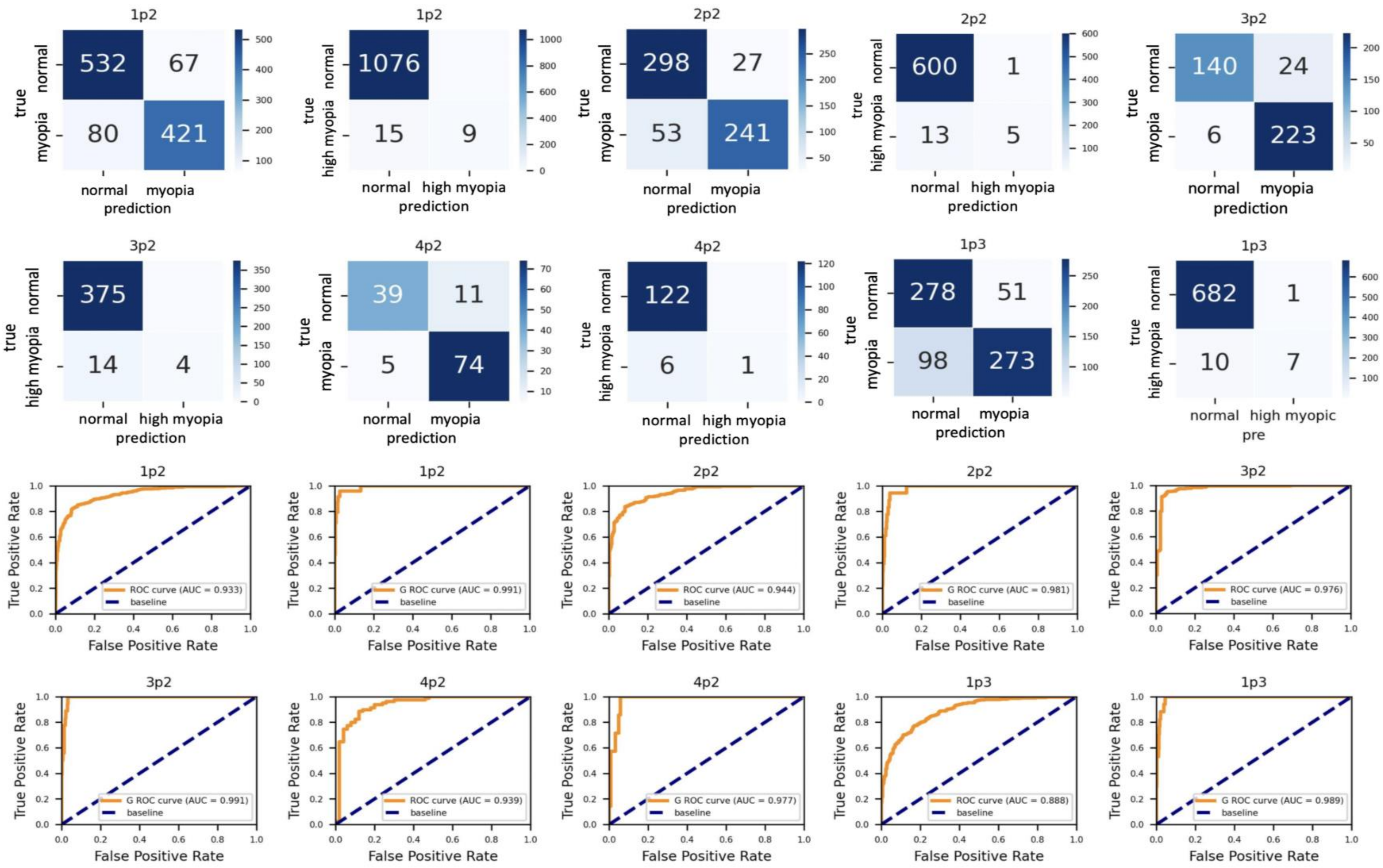

eFigure 5. Results of the Risk Prediction for Myopia and High Myopia.
Comprehensive visualization of model performance for binary classification tasks. Figures show ROC curves, confusion matrices, and performance metrics for predicting future myopia and high myopia risk across different prediction timeframes. Results demonstrate high predictive accuracy with AUC values exceeding 0.94 for myopia prediction and 0.98 for high myopia prediction.

© 2026 Kang M-T et al. *JAMA Network Open*

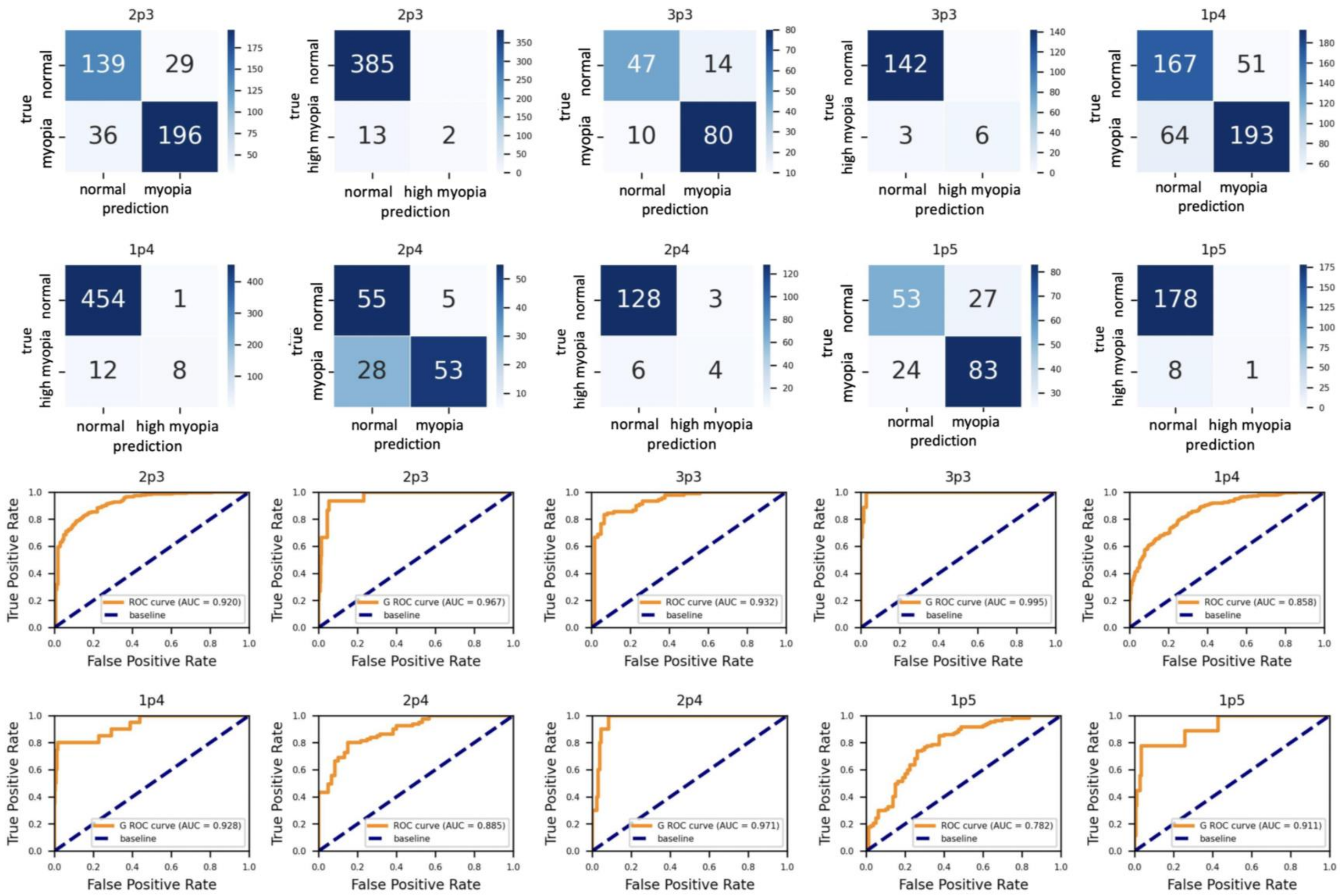

eFigure 5. *Continued*.
Comprehensive visualization of model performance for binary classification tasks. Figures show ROC curves, confusion matrices, and performance metrics for predicting future myopia and high myopia risk across different prediction timeframes. Results demonstrate high predictive accuracy with AUC values exceeding 0.94 for myopia prediction and 0.98 for high myopia prediction.

© 2026 Kang M-T et al. *JAMA Network Open*

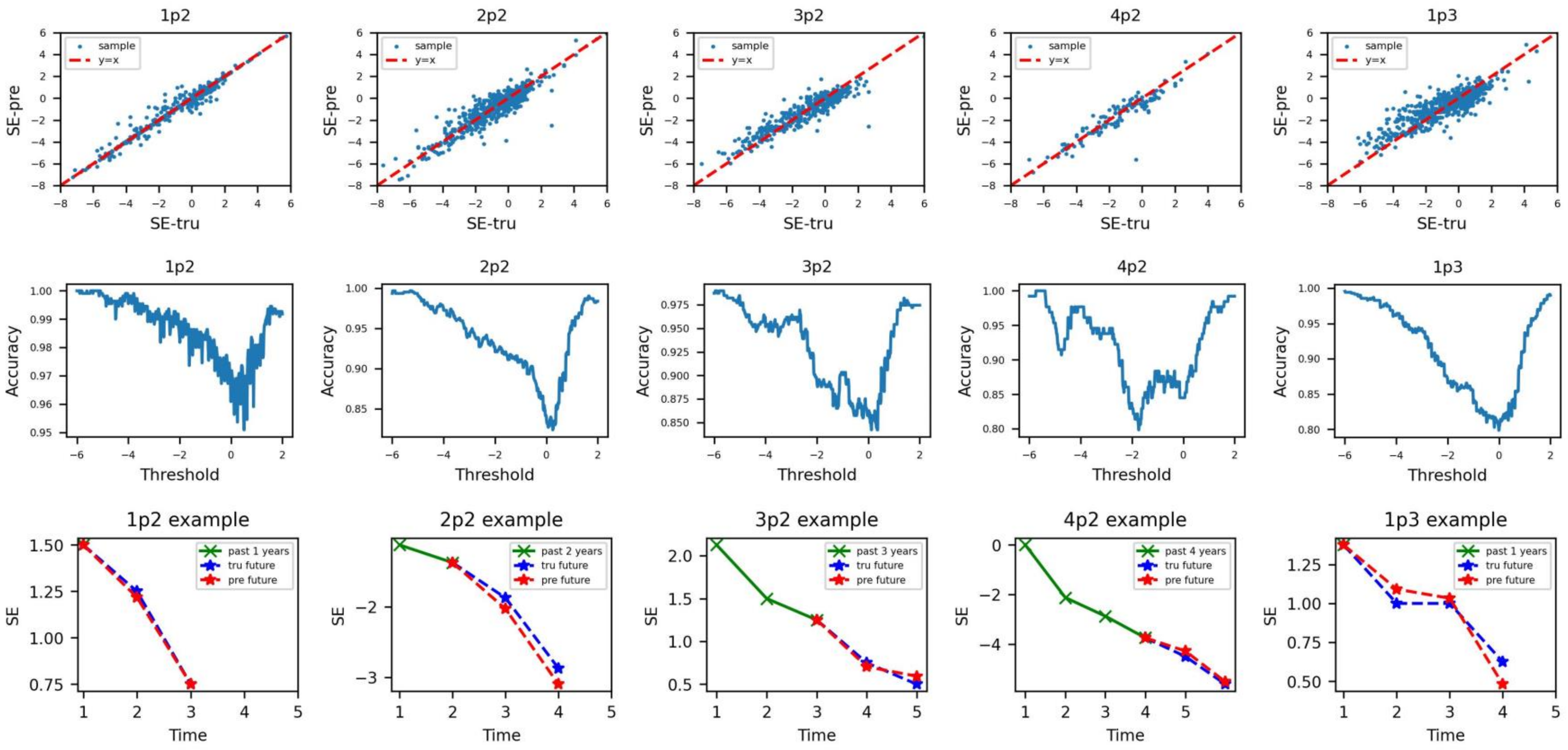

eFigure 6. Quantitative Prediction of Future Myopia Development Results.
Regression analysis results showing quantitative prediction of spherical equivalent refraction changes over time. Scatter plots compare predicted versus actual values, with correlation coefficients and mean absolute error metrics displayed. Line graphs demonstrate individual trajectory predictions compared to ground truth measurements, illustrating the model's capability for personalized myopia progression forecasting.

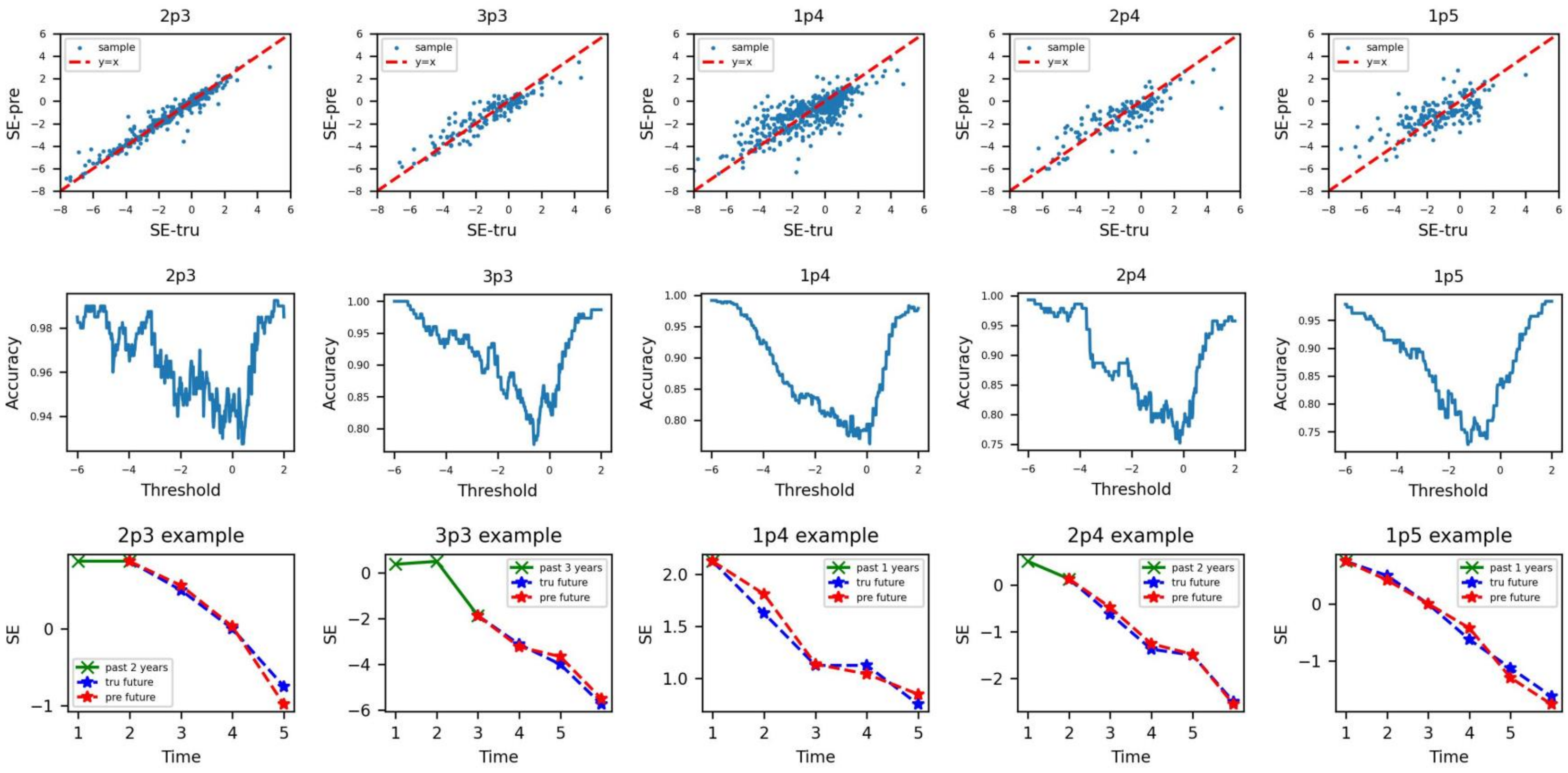

eFigure 6. *Continued.*

Regression analysis results showing quantitative prediction of spherical equivalent refraction changes over time. Scatter plots compare predicted versus actual values, with correlation coefficients and mean absolute error metrics displayed. Line graphs demonstrate individual trajectory predictions compared to ground truth measurements, illustrating the model's capability for personalized myopia progression forecasting.

© 2026 Kang M-T et al. *JAMA Network Open*

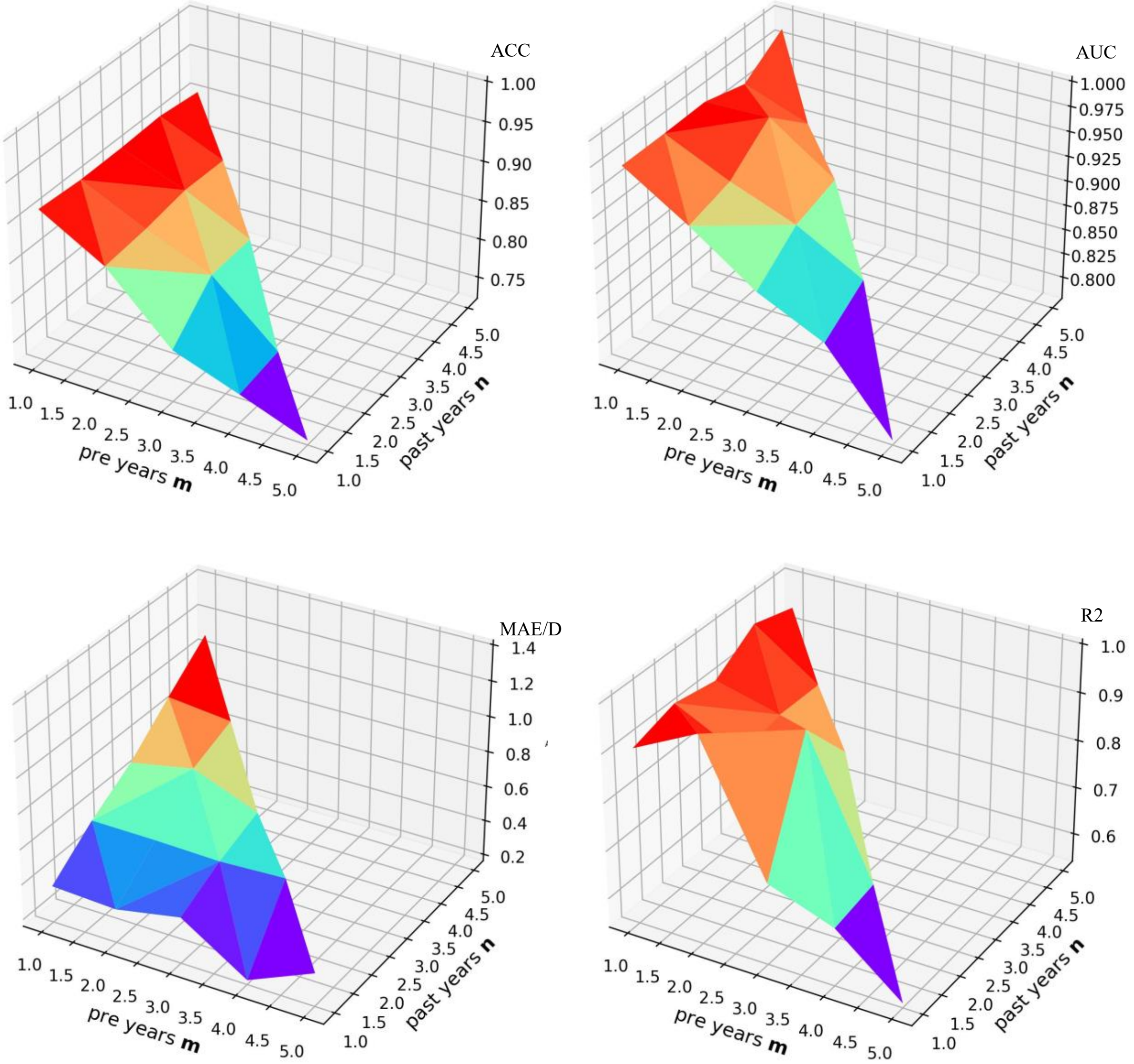

eFigure 7. Overall Performance Matrix of the nPm Model. Heatmap visualization showing performance transformation across different model configurations where n represents input sequence length and m represents prediction length. Color intensity indicates model performance metrics (AUC/MAE). Results demonstrate that prediction accuracy generally improves with longer input sequences (larger n) and decreases with longer prediction horizons (larger m), providing guidance for optimal model configuration selection in clinical applications.

© 2026 Kang M-T et al. *JAMA Network Open*

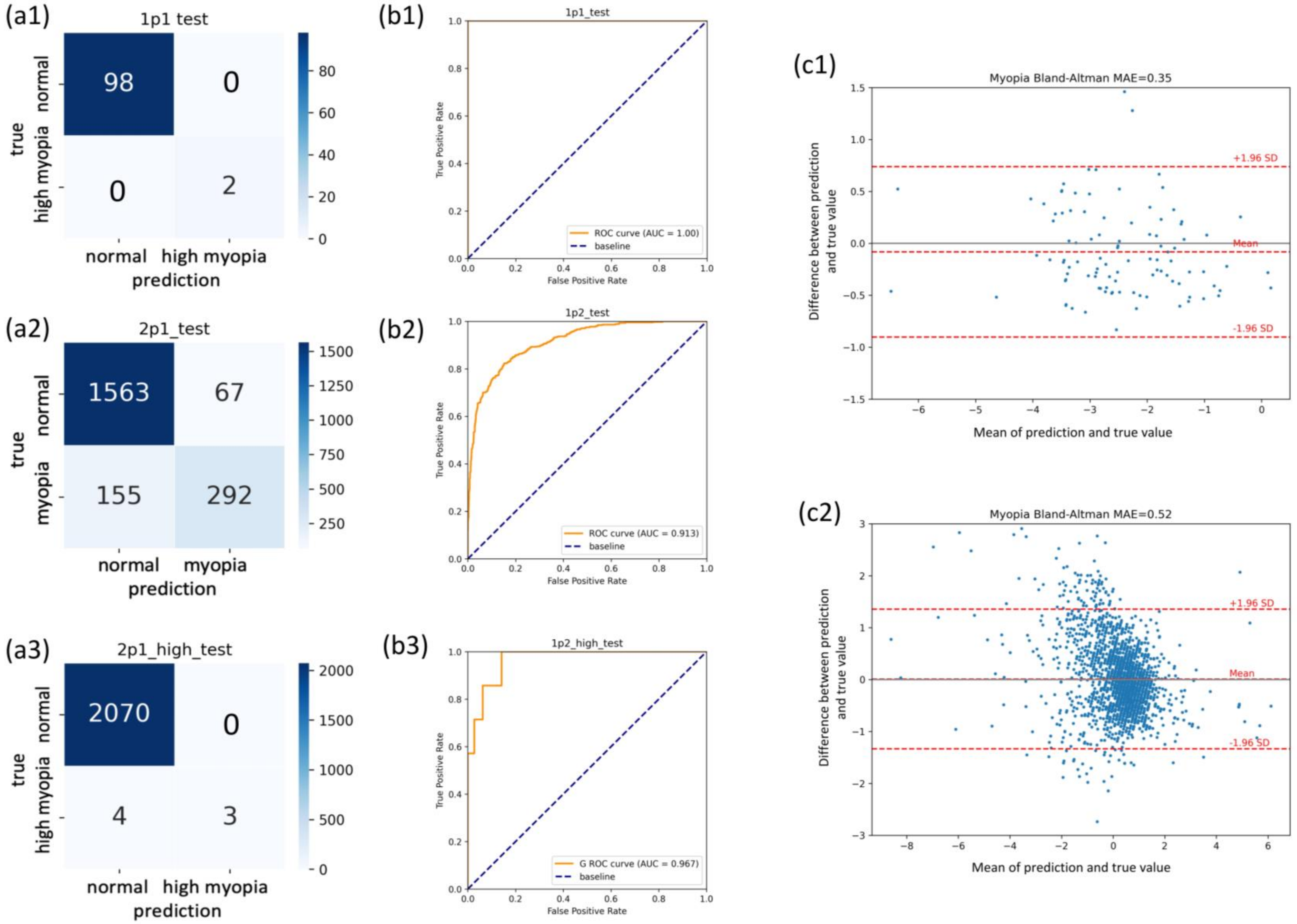

eFigure 8. External Validation Performance of Deep Learning Model Across Diverse Populations. External validation results demonstrating model generalizability across ethnically and geographically diverse pediatric populations. (a) Confusion matrices showing classification performance for high myopia prediction (1p1_test), myopia prediction (2p1_test), and high myopia prediction (2p1_high_test) respectively. (b) Corresponding ROC curves with AUC values of 1.00, 0.913, and 0.967 for the respective prediction tasks. (c) Bland-Altman plots demonstrating agreement between predicted and actual spherical equivalent refraction values, with mean absolute errors (MAE) of 0.35D and 0.52D respectively. A1,b1,c1 represent for Beijing cohort. A2,b2,c2,a3,b3,c3 represent for Lhasa cohort.

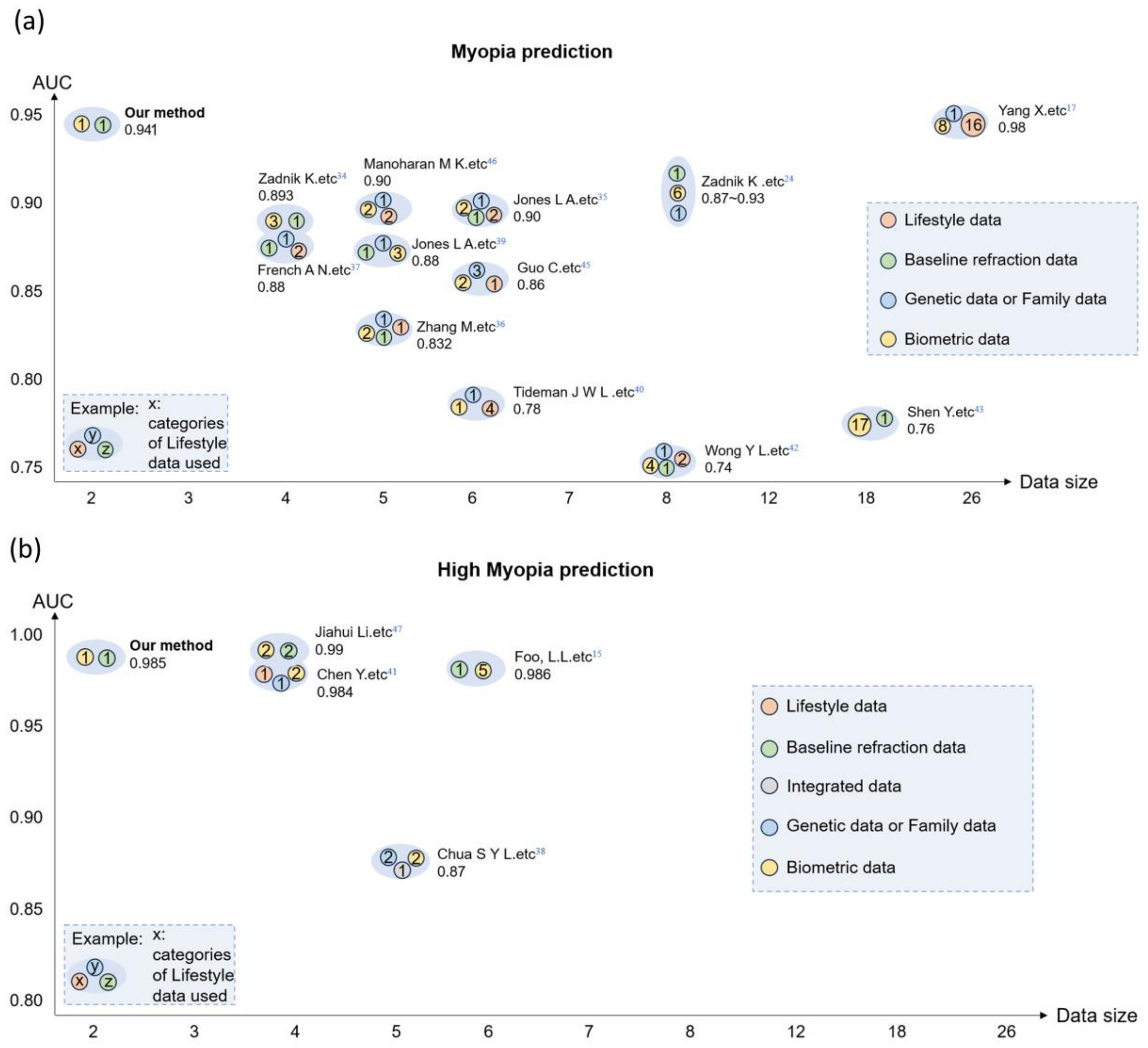

eFigure 9. Performance Comparison Across Myopia Prediction Studies. Performance comparison of deep learning approaches for (a) myopia prediction and (b) high myopia prediction. Bubble size represents sample size; Colour indicates data types (orange: lifestyle; green: baseline refraction; blue: genetic/family; yellow: biometric). Our method achieves superior AUC performance (0.941/0.985) with minimal data requirements compared to existing studies.

© 2026 Kang M-T et al. *JAMA Network Open*

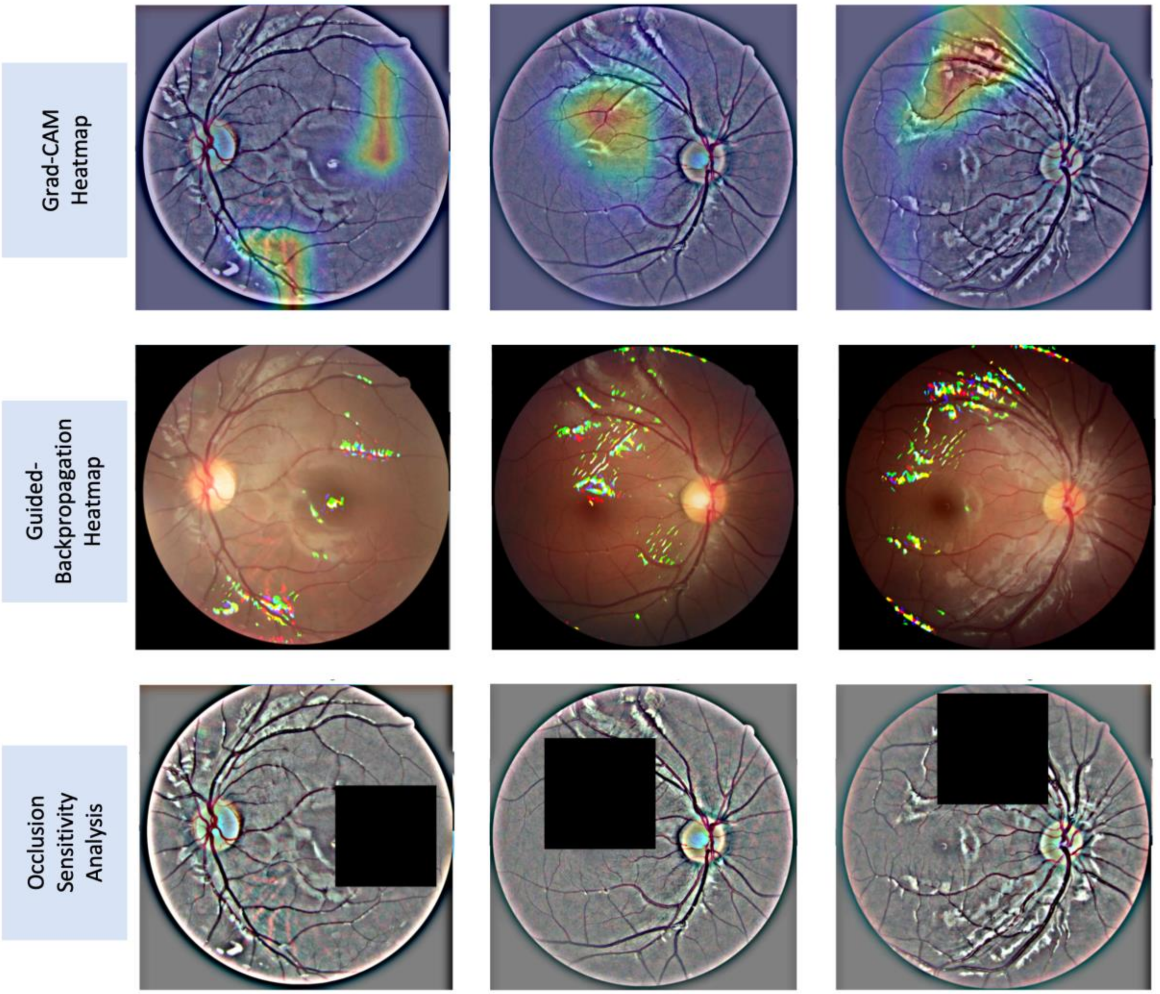

eFigure 10. Model Failure Case Analysis and Attention Visualization for Understanding Prediction Limitations. Representative examples of model misclassification cases with corresponding attention heatmaps and sensitivity analysis to identify failure patterns. Top row (Grad-CAM Heatmap): Class activation mapping showing regions of highest model attention during incorrect predictions, with warm colors (red/yellow) indicating areas of peak neural network focus and cool colors (blue/purple) representing lower attention regions. Middle row (Guided Backpropagation Heatmap): Fine-grained feature attribution maps highlighting specific retinal structures and vessel patterns that contributed to erroneous predictions, with green markers indicating key decision-influencing pixels. Bottom row (Occlusion Sensitivity Analysis): Systematic masking analysis revealing critical fundus regions whose occlusion significantly alters prediction confidence, with black squares representing occluded areas that cause prediction changes.

© 2026 Kang M-T et al. *JAMA Network Open*
