# Supplementary material for: Deep Learning Prediction of Childhood Myopia Progression Using Fundus Image and Refraction Data
Source: JAMA Netw Open. 2026 Jan 26;9(1):e2553543. doi: 10.1001/jamanetworkopen.2025.53543 (PMC12836131; doi:10.1001/jamanetworkopen.2025.53543)
Supplement: Supplement 2. — Data Sharing Statement [file jamanetwopen-e2553543-s002.pdf]

Data Sharing Statement

Kang. Deep Learning Prediction of Childhood Myopia Progression Using Fundus Image and Refraction Data. *JAMA Netw Open*. Published January 23, 2026.
doi:10.1001/jamanetworkopen.2025.53543

Data
Data available: Yes
Data types: Data (not involving human participants), Data dictionary
How to access data: lishiming81@163.com
When available: With publication

Supporting Documents
Document types: Statistical/analytic code, Informed consent form
How to access documents: lishiming81@163.com
When available: With publication

Additional Information
Who can access the data: Shi-Ming Li
Types of analyses: For specified purpose
Mechanisms of data availability: After approval of a proposal
